# Clinicopathological analysis of primary intestinal diffuse large B‐cell lymphoma: Prognostic evaluation of CD5, PD‐L1, and Epstein‐Barr virus on tumor cells

**DOI:** 10.1002/cam4.1875

**Published:** 2018-11-18

**Authors:** Eri Ishikawa, Seiichi Kato, Kazuyuki Shimada, Tsutomu Tanaka, Yuka Suzuki, Akira Satou, Kei Kohno, Ayako Sakakibara, Takeshi Yamamura, Masanao Nakamura, Ryoji Miyahara, Hidemi Goto, Shigeo Nakamura, Yoshiki Hirooka

**Affiliations:** ^1^ Department of Gastroenterology and Hepatology Nagoya University Graduate School of Medicine Nagoya Japan; ^2^ Department of Pathology and Laboratory Medicine Nagoya University Hospital Nagoya Japan; ^3^ Department of Pathology and Molecular Diagnostics Aichi Cancer Center Hospital Nagoya Japan; ^4^ Department of Hematology and Oncology Nagoya University Graduate School of Medicine Nagoya Japan; ^5^ Department of Endoscopy Aichi Cancer Center Hospital Nagoya Japan; ^6^ Department of Pathology Aichi Medical University Hospital Nagakute Japan; ^7^ Department of Endoscopy Nagoya University Hospital Nagoya Japan

**Keywords:** diffuse large B‐cell lymphoma, Epstein‐Barr virus, PD‐L1, primary intestinal lymphoma, rituximab

## Abstract

**Background:**

Primary intestinal diffuse large B‐cell lymphoma (iDLBCL) is rare. In this study, we investigated the clinicopathological features of this disease to further understand the prognostic value of CD5, programmed cell death ligand 1 (PD‐L1), and Epstein‐Barr virus (EBV) on tumor cells.

**Methods:**

Tumor specimens from 62 patients consecutively diagnosed with primary iDLBCL at a single institution were analyzed.

**Results:**

Our series consisted of EBV‐positive (EBV^+^) iDLBCL (n = 10), de novo CD5^+^ iDLBCL (n = 4), and DLBCL, not otherwise specified (DLBCL‐NOS; n = 48). Notably, seven of 10 EBV^+^ cases had treated lymphoma‐associated (n = 4) or iatrogenic immunodeficiency (n = 3). Two of 10 EBV^+^ cases expressed PD‐L1 on tumor cells, whereas the remaining eight were positive for PD‐L1 on microenvironment immune cells. Only one DLBCL‐NOS case had neoplastic PD‐L1 expression with a giant cell‐rich appearance. Both EBV‐harboring and PD‐L1 expression on tumor cells, but not CD5, were associated with worse overall survival (OS) in iDLBCL patients receiving rituximab‐containing chemotherapy (*P* = 0.0354, *P* = 0.0092, and *P* = 0.1097, respectively). Multivariate analysis identified PD‐L1 positivity on tumor cells (*P* = 0.0106), PD‐L1 negativity on microenvironment immune cells (*P* = 0.0193), and EBV positivity (*P* = 0.0324) as poor independent prognostic factors for OS. Among iDLBCL cases without any EBV association, CD5 positivity, or neoplastic PD‐L1 expression, high PD‐L1 expression (≥40%) on microenvironment immune cells predicted an extremely favorable outcome.

**Conclusion:**

EBV^+^ iDLBCL mainly comprised immunodeficiency‐associated patients, which may highlight the specificity of the intestine. PD‐L1 expression on tumor cells or microenvironment immune cells was found to have an opposite prognostic impact in iDLBCL.

## 1 INTRODUCTION

The gastrointestinal tract is the most common (34%‐44%) extranodal site of involvement in diffuse large B‐cell lymphoma (DLBCL).^1^, ^2^ Small and large intestinal lymphoma behave differently than gastric lymphoma, with comparatively lower survival and account for approximately 25% of gastrointestinal DLBCL cases.^3^, ^4^ DLBCL patients with small intestine involvement have significantly worse overall survival (OS) and often require a combination of chemotherapy and surgery due to increased complication rates and decreased survival.^5^, ^6^

Epstein‐Barr virus (EBV) was the first oncogenic virus identified and is associated with a number of lymphoid malignancies, including Burkitt lymphoma, DLBCL, and iatrogenic immunodeficiency‐associated lymphoproliferative disorders (LPDs).^7^, ^8^ The 2017 WHO classification of malignant lymphoma encompassed these diverse diseases and emphasized that EBV‐positive (EBV^+^) DLBCL, not otherwise specified (DLBCL‐NOS), often affects both young and elderly immunocompetent patients. An increasing number of reports provide additional evidence to support this assertion,^9^, ^10^ though their incidence and prognostic significance remain controversial. EBV^+^ DLBCL affects extranodal sites in approximately two‐thirds of elderly immunocompetent patients.^9^ We previously reported that EBER positivity is associated with an adverse outcome in patients with primary gastric DLBCL.^14^ However, the issue of tumor cells harboring EBV has not been addressed in primary intestinal DLBCL (iDLBCL) due to diagnostic difficulties. The advent of double‐balloon endoscopy was a significant breakthrough for the visual diagnosis of diseases located deep in the small intestine and enabled us to analyze this rare disease of primary iDLBCL.^15^

The programmed cell death (PD)‐1 pathway has become an attractive therapeutic target in multiple cancers.^16^, ^17^ Blocking the interaction between PD‐1 and its ligands, PD‐L1 and PD‐L2, leads to impressive antitumor responses and clinical benefit in a subset of patients,^18^, ^19^ including relapsed and refractory DLBCL.^20^, ^21^ However, predicting tumor responses to PD‐1 blockade remains a major challenge. Recent studies have described a correlation between PD‐L1 expression and an improved response to PD‐1 blockade in patients with multiple types of cancer.^22^, ^23^ PD‐L1 expression is also considered a hallmark of EBV‐associated LPDs, including EBV^+^ plasmablastic lymphoma, EBV^+^ post‐transplant LPDs, and EBV^+^ DLBCL‐NOS.^12^, ^17^, ^25^ This frequent upregulation of PD‐L1 in EBV^+^ lymphoma patients has been the focus in the era of checkpoint inhibition.

Recently, large cohort studies have shown a higher incidence of extranodal disease including central nervous system (CNS) involvement and adverse outcome in patients with CD5^+^ DLBCL.^26^, ^27^ However, involvement of the gastrointestinal tract has been reported to occur less frequently in CD5^+^ DLBCL than CD5^−^ DLBCL.^28^ Therefore, the prognostic significance of CD5 positivity in primary iDLBCL has not been well analyzed.

In the present study, we investigated 62 cases of primary iDLBCL to further understand the clinicopathological features and biological properties of this rare disease by surveying the association of EBV and the expression of CD5 and PD‐L1.

## 2 MATERIALS AND METHODS

### 2.1 Patient selection

This retrospective study included data from 62 patients with primary iDLBCL diagnosed between 2004 and 2017 at Nagoya University Hospital and 18 affiliated institutions, comprising 34 surgical excision specimens and 28 endoscopic biopsy specimens. Diagnosis was established according to histopathological and immunohistochemical criteria, based on the 2017 WHO classification system. All cases satisfied the criteria for primary gastrointestinal lymphoma as defined by Lewin et al.^29^ The best method for discriminating primary intestinal DLBCL from systemic DLBCL involving the intestine is not clear. Lymphoma at the intestine was considered primary if the main bulk of disease is located in the intestine. Patients with impairment of the immune system secondary to primary immunodeficiency, previous solid cancer, or lymphoma were included. The clinical stage was evaluated according to the Lugano classification for gastrointestinal non‐Hodgkin lymphoma.^30^ The type of lymphoma was classified macroscopically as polypoid, ulcerative, lymphomatous polyposis, diffuse‐infiltrating, or mixed by three experienced endoscopists (EI, TY, and MN) based on the endoscopic findings in 43 cases and on the surgical excision specimens in five cases.^31^ All pathology specimens were reviewed by EI, KK, and SN. The study was approved by the Institutional Review Board of Nagoya University.

### 2.2 Immunohistochemistry and in situ hybridization studies

Tissue samples were fixed in 10% formalin, embedded in paraffin, and 5‐μm‐thick sections stained with hematoxylin and eosin. The monoclonal antibodies used for immunohistochemistry are listed in Table S1. Lymphoid cell staining was considered positive for PD‐L1 (nPD‐L1^+^) when ≥5% of the neoplastic lymphoid cells demonstrated moderate or strong membrane staining with a PD‐L1 specific antibody (clone SP142). A case was considered positive for PD‐L1 in the microenvironment (miPD‐L1^+^) when, among the total tissue cellularity, ≥20% comprised nonmalignant cells with moderate or strong membrane or cytoplasmic PD‐L1‐specific staining. The threshold used here is comparable to that from a prior publication involving the same clone antibody.^32^, ^33^ The percentage of PD‐L1‐positive stromal cells was determined independently by three (EI, KK, and SN) and any discrepancies discussed using the multi‐headed microscope. All cases were tested for EBV‐encoded small RNA (EBER) using in situ hybridization (ISH) as described previously.^9^ Cases were considered EBER‐positive when nuclear expression of EBER was observed in ≥80% of tumor cells. Formalin‐fixed paraffin‐embedded (FFPE) tissue sections were used for dual‐color FISH analysis using a SPEC CD274, PDCD1LG2/CEN9 Dual Color Probe (Zytovision, Bremerhaven, Germany).

### 2.3 Statistical analysis

Correlations between two groups were determined using the Fisher's exact test and Mann‐Whitney *U* test. The survival distribution was estimated by the Kaplan‐Meier method, and groups were compared by the log‐rank test. Univariate Cox regression analyses were performed to assess the effects of prognostic factors. The multivariate analysis was performed using a backward stepwise method, and *P* < 0.1 was the threshold for inclusion in the model. All statistical analyses were performed with EZR, a graphical user interface for R (The R Foundation for Statistical Computing, Vienna, Austria).^34^

## 3 RESULTS

### 3.1 Clinicopathological characteristics of iDLBCL

The study cohort included 62 patients: 42 males and 20 females (male:female ratio = 2.1:1), with a median age of 68 years (range, 15‐88 years). The clinical data are summarized in Table 1. Twenty‐two (36%) of 62 patients had primary tumors in the ileocecum, 13 (21%) in the jejunum, and 10 (16%) in the large intestine. Eleven (18%) patients had intestinal obstruction, 7 (12%) with perforation, and 13 (23%) with performance status (PS) 2‐4. Lugano stage was known for 59 cases; 5 (8%) were in Lugano stage I, 11 (19%) in stage II1, 15 (25%) in stage II2, 7 (12%) in stage IIE, and 21 (36%) in stage IV. Fourteen (25%) of 56 patients available in their full data had an International Prognostic Index (IPI) of high‐intermediate/high (HI/H). Macroscopically, 35 (73%) of 48 evaluated cases were classified as ulcerative type, 10 (21%) as polypoid type, and three (6%) as diffuse‐infiltrating or others. Thirteen (22%) had multiple intestinal lesions and two (5%) of 41 patients who underwent esophagogastroduodenoscopy at diagnosis had gastric involvement. All cases enrolled in this study exhibited a predominant proliferation of medium‐to‐large lymphoid cells without evidence of concomitant low‐grade lesions. Fifty‐seven (92%) cases were CD20^+^, whereas the remaining five cases were positive for CD79a or PAX5 in the absence of CD20. CD5 and CD10 were detected in 6 (10%) and 22 (36%) cases, respectively.

**Table 1:** Clinicopathological characteristics of primary intestinal DLBCL patients

Characteristics	Total		EBV‐positive		EBV‐negative		*P* a
	n = 62	n[%]	n = 10	n[%]	n = 52	n[%]	
Sex (male/female)	42/20	2.1	4/6	0.7	38/14	2.8	0.063
Age (y), median (range)	68	15‐88	74	47‐82	67	15‐88	0.18
Age > 60 y	46/62	74	8/10	80	38/52	73	1.00
Primary site							
Duodenum	5/62	8	2/10	20	3/52	6	0.42
Jejunum	13/62	21	1/10	10	12/52	23	
Ileum	8/62	13	0/10	0	8/52	15	
Ileocecum	22/62	36	4/10	40	18/52	35	
large intestine	10/62	16	2/10	20	8/52	15	
Multiple sites	4/62	6	1/10	10	3/52	6	
Macroscopic type							
Ulcerative	35/48	73	7/9	78	28/39	72	1.00
Polypoid	10/48	21	2/9	22	8/39	20	
Diffuse‐infiltrating or others	3/48	6	0/9	0	3/39	8	
Multiple intestinal lesions	13/59	22	5/10	50	8/49	16	0.033
Bulky mass present	10/59	17	2/9	22	8/50	16	0.64
Gastric involvement	2/41	5	0/7	0	2/34	6	1.00
Abdominal pain	35/60	58	6/10	60	29/50	58	1.00
Intestinal obstruction	11/60	18	0/10	0	11/50	22	0.18
Perforation	7/59	12	2/10	20	5/49	10	0.34
PS 2‐4	13/57	23	5/9	56	8/48	17	0.022
Extranodal sites > 1	12/60	20	2/10	20	10/50	20	1.00
B symptoms present	11/55	20	3/9	33	8/46	17	0.36
Serum LDH > normal	17/59	29	4/10	40	13/49	27	0.45
sIL‐2R ≥ 1000 U/mL	27/55	49	6/8	75	21/47	45	0.14
Lugano stage II2/IIE/IV	43/59	73	9/10	90	34/49	69	0.26
IPI High‐int, High	14/56	25	6/9	67	8/47	17	0.0050
Immunophenotype							
CD5	6/59	10	2/9	22	4/50	8	0.22
CD10	22/61	36	1/10	10	21/51	41	0.079
CD20	57/62	92	8/10	80	49/52	94	0.18
CD30	2/33	6	2/5	40	0/28	0	0.019
BCL‐2	38/61	62	4/10	40	34/51	67	0.16
nPD‐L1 (≥5%)	3/59	5	2/10	20	1/49	2	0.072
miPD‐L1 (≥20%)	39/56	70	8/8	100	31/48	65	0.090
non‐GCB immunophenotype	38/60	63	9/10	90	29/50	58	0.076
Treatment							
R‐containing CTx	51/59	86	9/10	90	42/49	86	1.00
R‐CTx	20/51	39	4/9	44	16/42	38	1.00
R‐CTx+Surgery	30/51	59	5/9	56	25/42	60	1.00
R‐CTx+Rad	1/51	2	0/9	0	1/42	2	1.00
No. of cycles, median (range)	6	1‐8	6	2‐8	6	1‐8	0.43
Surgery alone	2/59	3	0/10	0	2/49	4	1.00
No treatment	4/59	7	1/10	10	3/49	6	0.53
Treatment response (R‐containing CTx)							
CR	37/51	73	6/9	67	31/42	74	0.69
PR	5/51	10	2/9	22	3/42	7	0.21
SD or PD	9/51	18	1/9	11	8/42	19	1.00

CTx, chemotherapy; CR, complete remission; GCB, germinal center B‐cell; IPI, International Prognostic Index; LDH, lactate dehydrogenase; miPD‐L1, microenvironmental programmed cell death ligand 1; nPD‐L1, neoplastic programmed cell death ligand 1; PD, progressive disease PR, partial remission; PS, performance status; R, rituximab; SD, stable disease; sIL‐2R, soluble interleukin‐2 receptors.

a *P* value are for the comparison of EBV‐positive and EBV‐negative primary intestinal DLBCL patients.

### 3.2 Clinical course of iDLBCL

Fifty‐one (86%) of 59 patients with primary iDLBCL given treatment information received multi‐agent chemotherapy combined with rituximab. Of these, the most common regimen was rituximab, cyclophosphamide, doxorubicin, vincristine, and prednisolone (38/51, 75%). Twenty (39%) of 51 were treated with rituximab‐containing chemotherapy alone, 30 (59%) underwent surgical resection initially, and one (2%) received additional irradiation. Twelve cases presented with a need for emergency surgery due to perforation, obstruction, or fistula. Among 51 iDLBCL patients treated with rituximab‐containing chemotherapy, 37 (73%) achieved complete remission (CR) and 7 (14%) developed progressive disease (PD). The 3‐year progression‐free survival (PFS) and overall survival (OS) rates were 63% and 73%, respectively, with a median follow‐up of 42 months (range 3.5‐150 months). The PFS and OS rates were significantly greater in patients with Lugano stage I/II1 than in patients with Lugano stage II2/IIE/IV (3‐year PFS: 100% vs 50%; *P* = 0.00082, 3‐year OS: 100% vs 63%; *P* = 0.0076).

### 3.3 Clinicopathological characteristics of EBV‐positive iDLBCL and de novo CD5‐positive iDLBCL

Our series consisted of EBV^+^ iDLBCL (n = 10), de novo CD5^+^ iDLBCL (n = 4), and DLBCL‐NOS cases (n = 48). EBV‐harboring on ≥80% of their tumor cells was detected in 10 (16%) patients by EBER‐ISH. Surprisingly, seven of these were related with treated lymphoma‐associated (peripheral T‐cell lymphoma [n = 2], classic Hodgkin lymphoma [n = 2]) or iatrogenic immunodeficiency (methotrexate [n = 1], infliximab [n = 1], and tacrolimus [n = 1], Table 2). The other one also had a synchronous gastric carcinoma, while the remaining two had no episode suggestive of immunodeficiency in their lifestyle analysis. This prompted us to reexamine the presence of events related to immunodeficiency among EBV^−^ iDLBCL cases, but none were found. EBV latency II (LMP1^+^, ENBA2^‐^) and III (LMP1^+^, EBNA2^+^) each were found in three patients.

**Table 2:** Presentation, treatment, and outcome of patients with EBV^+,^ CD5^+^ and/or nPD‐L1^+^ intestinal DLBCL (n = 15)

No	Age	Sex	Primary site	Source of immunosuppression	EBV	CD5	nPD‐L1	Treatment	Response	Time to relapse (mo)	Status	Length of follow‐up (mo)
1	63	M	Ileocecum	Old age	−	−	+(100%)	R‐CTx+S	CR	5	DD	12
2	75	F	A colon	Old age	+	−	+(50%)	R‐CTx	PR	6	DD	10
3	74	F	Ileocecum	PTCL	+	−	+(20%)	R‐CTx+S	CR	22	DD	26
4	70	F	Ileocecum	PTCL	+	−	−	R‐CTx	CR	11	DD	45
5	82	F	Duodenum	cHL	+	−	−	NT			DOC	0.3
6	80	M	Jejunum	cHL	+	+	−	R‐CTx+S	PD		DD	11
7	82	F	Ileocecum	MTX	+	+	−	R‐CTx+S	CR		DOC	80
8	74	M	Rectum	Infliximab	+	−	−	R‐CTx	CR	52	DD	61
9	47	F	Duodenum	Tacrolimus	+	−	−	R‐CTx	PR		AWD	4
10	57	M	Jejunum	Synchronous GC	+	−	−	R‐CTx+S	CR		NED	41
11	66	M	Ileocecum	Old age	+	−	−	R‐CTx+S	CR		NED	35
12	71	M	Jejunum	RC	−	+	−	R‐CTx+S	PR		DOC	9
13	64	M	Jejunum	Old age	−	+	−	R‐CTx+S	CR		NED	98
14	76	M	Jejunum	Old age	−	+	−	R‐CTx	CR	48	AWD	54
15	73	F	Jejunum	Old age	−	+	−	R‐CTx	PD		DD	4

A colon, ascending colon; AWD, alive with disease; cHL, classic Hodgkin lymphoma; CR, complete remission; CTx, chemotherapy; DD, died of disease; DOC, died of other causes; F, female; GC, gastric carcinoma; M, male; MTX, methotrexate; NED, no evidence of disease; nPD‐L1, neoplastic programmed cell death ligand 1; NT, no treatment; PD, progressive disease; PR, partial remission; PTCL, peripheral T‐cell lymphoma; R, rituximab; RC, renal carcinoma; S, surgery.

Compared with EBV^−^ iDLBCL, EBV^+^ cases had a higher rate of CD30 positivity (40% vs 0%, *P* = 0.019), PS 2‐4 (56% vs 17%, *P* = 0.022), multiple intestinal lesions (50% vs 16%, *P* = 0.033), IPI HI/H (67% vs 17%, *P* = 0.0050), and non‐germinal center B‐cell (GCB) immunophenotype (90% vs 58%, *P* = 0.076). PD‐L1 expression on tumor cells was observed in two (20%) of 10 EBV^+^ iDLBCL cases, which was higher than in EBV^−^ cases (2% [1/49], *P* = 0.072). The remaining eight EBV^+^ cases were positive for PD‐L1 on microenvironment immune cells, with a higher rate than in EBV^−^ iDLBCL (100% vs 65%, *P* = 0.090).

**Figure 1:**
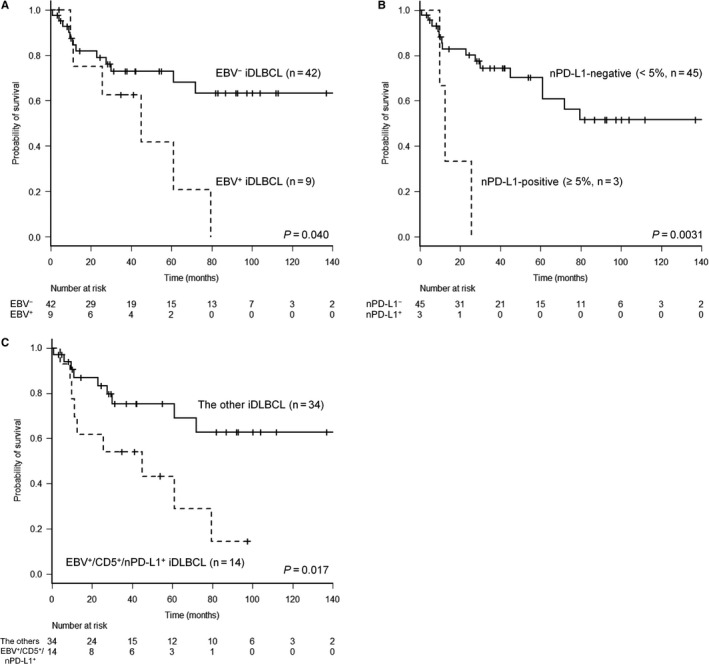
Kaplan‐Meier curves of patients with intestinal DLBCL (iDLBCL) treated with rituximab‐containing chemotherapy. A, Overall survival (OS) according to EBV status on tumor cells. B, OS according to neoplastic PD‐L1 expression. C, A comparison of survival between EBV^+^, CD5^+^, and/or nPD‐L1^+^ iDLBCL cases and the other iDLBCLs

Among the iDLBCL patients receiving rituximab‐containing chemotherapy, EBV^+^ iDLBCL had significantly worse OS than EBV^−^ iDLBCL (63% vs 73% for 3‐year OS, *P* = 0.040, Figure 1A).

Four cases of de novo CD5^+^ iDLBCL were found in our series, all having primary tumors in the jejunum and bearing common (monomorphic) appearance without neoplastic PD‐L1 expression but failed to show the prognostic inferiority (*P* = 0.1097) because of the paucity of the enrolled cases.

### 3.4 Expression of PD‐L1 on tumor cells in patients with iDLBCL

**Figure 2:**
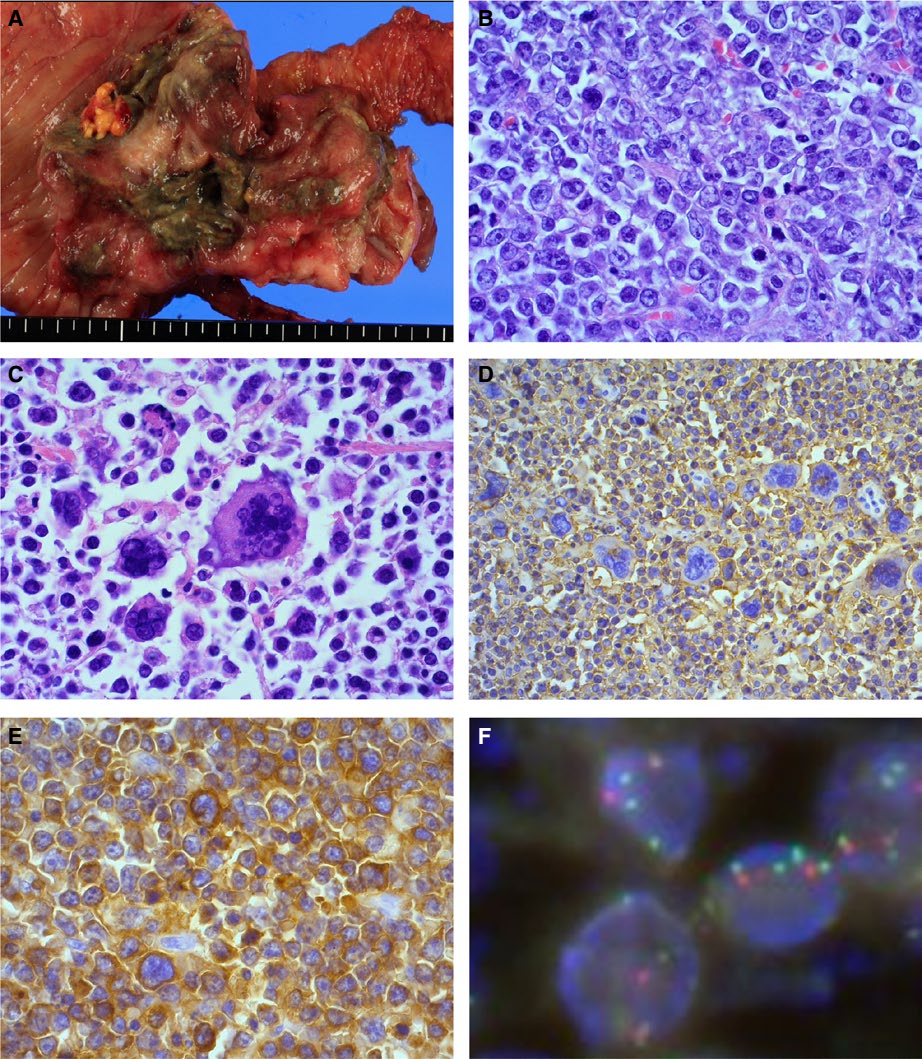
nPD‐L1‐positive iDLBCL (case #1). A, Surgical excision specimen shows an ulcerative tumor in the ileocecum. B, Diffuse lymphoid proliferation of medium‐to‐large cells is identified. C, Giant cell‐rich appearance is observed. D, The tumor cells are positive for CD20. E, PD‐L1 expression on 100% of tumor cells is seen. F, Fluorescence in situ hybridization (FISH) for PD‐L1 shows amplification of the PD‐L1 gene locus

Three (5%; case #1‐3) of 59 examined cases had PD‐L1 expression on tumor cells with ages of onset of 63, 75, and 74 years, consisting of one iDLBCL‐NOS and two EBV^+^ iDLBCLs described above (Table 2). In two cases (case #1 and 3) with ileocecal involvement, the proportion of PD‐L1‐positive tumor cells in surgical excision specimens was 100% and 20%, respectively. Case #1 was unique in partially having giant cell‐rich appearance, which has been documented as a morphologic variant in de novo CD5^+^ DLBCL, despite its CD5 negativity on the FFPE section (Figure 2). In case #1, the CD274/PD‐L1 gene copy number status was also assessed by fluorescence in situ hybridization and gene amplification detected. On the other hand, the remaining one (case #2) had ascending colon involvement, and endoscopic biopsy specimens presented PD‐L1 expression on 50% of tumor cells. These three patients received rituximab‐containing chemotherapy and achieved CR or PR, but relapsed (5, 6, and 22 months) and died of disease (12, 10, and 26 months). nPD‐L1‐positive patients had significantly inferior OS compared to nPD‐L1‐negative cases (*P* = 0.0031, Figure 1B) with a 3‐year OS of 0% and 74%, respectively. PFS was also significantly different (*P* = 0.0084).

### 3.5 Prognostic factors of iDLBCL in the rituximab era

The clinical factors associated with worse OS in univariate Cox analysis were as follows: IPI HI/H (*P* = 0.0005), PS 2‐4 (*P* = 0.0052), nPD‐L1 positivity (*P* = 0.0092), and EBER positivity (*P* = 0.0354, Table 3). Multivariate analysis with nPD‐L1 expression and other factors revealed that nPD‐L1 positivity (≥5%) is a significant prognostic factor for OS (*P* = 0.0106). A similar multivariate analysis with miPD‐L1 expression identified miPD‐L1 negativity (<20%, *P* = 0.0193) and EBER positivity (*P* = 0.0324) as poor independent prognostic factors of OS.

**Table 3:** Univariate and multivariate analysis of OS in primary intestinal DLBCL in the rituximab era (n = 51)

	Univariate		Multivariate^a^		Multivariate^b^	
	HR (95% CI)	*P*	HR (95% CI)	*P*	HR (95% CI)	*P*
Male	0.72 (0.27‐1.95)	0.5209				
Age > 60 y	1.67 (0.54‐5.15)	0.3683		—		—
Except for ulcerative type	1.60 (0.48‐5.35)	0.4439				
Multiple intestinal lesions	2.06 (0.71‐5.98)	0.1832				
Bulky mass	1.96 (0.68‐5.67)	0.2133				
Abdominal pain	4.09 (0.93‐17.9)	0.0617		—		—
Perforation	2.07 (0.67‐6.42)	0.2077				
PS 2‐4	3.94 (1.51‐10.3)	0.0052		—		—
Extranodal sites > 1	1.96 (0.69‐5.57)	0.209				
B symptoms present	1.20 (0.39‐3.70)	0.7483				
Serum LDH > normal	2.06 (0.75‐5.64)	0.1603				
sIL‐2R ≥ 1000 U/mL	2.36 (0.90‐6.25)	0.0825		—		—
Lugano stage II2/IIE/IV	14.0 (0.77‐253)	0.0741		—		—
IPI High‐int, High	6.23 (2.22‐17.5)	0.0005				
Immunophenotype						
CD5 positive	2.52 (0.81‐7.84)	0.1097				
CD20 negative	2.60 (0.58‐11.6)	0.2108				
BCL‐2 positive	0.87 (0.33‐2.26)	0.7731				
nPD‐L1 positive (≥5%)	5.92 (1.55‐22.5)	0.0092	5.72 (1.50‐21.8)	0.0106		
miPD‐L1 negative (<20%)	2.74 (0.99‐7.62)	0.0531			4.36 (1.27‐15.0)	0.0193
Non‐GCB type	0.98 (0.38‐2.53)	0.9615				
EBER positive	2.95 (1.08‐8.07)	0.0354		—	4.56 (1.14‐18.3)	0.0324
R‐CTx alone	1.54 (0.59‐4.01)	0.3759				

CTx, chemotherapy; EBER, EBV‐encoded small RNA; IPI, International Prognostic Index; LDH, lactate dehydrogenase; miPD‐L1, microenvironmental programmed cell death ligand 1; non‐GCB, non‐germinal center B‐cell; nPD‐L1, neoplastic programmed cell death ligand 1; OS, overall survival; PS, performance status; R, rituximab; sIL‐2R, soluble interleukin‐2 receptors.

a Multivariate analysis with nPD‐L1 expression and other factors.

b Multivariate analysis with miPD‐L1 expression and other factors.

### 3.6 Expression of PD‐L1 on microenvironment immune cells in patients with iDLBCL

**Figure 3:**
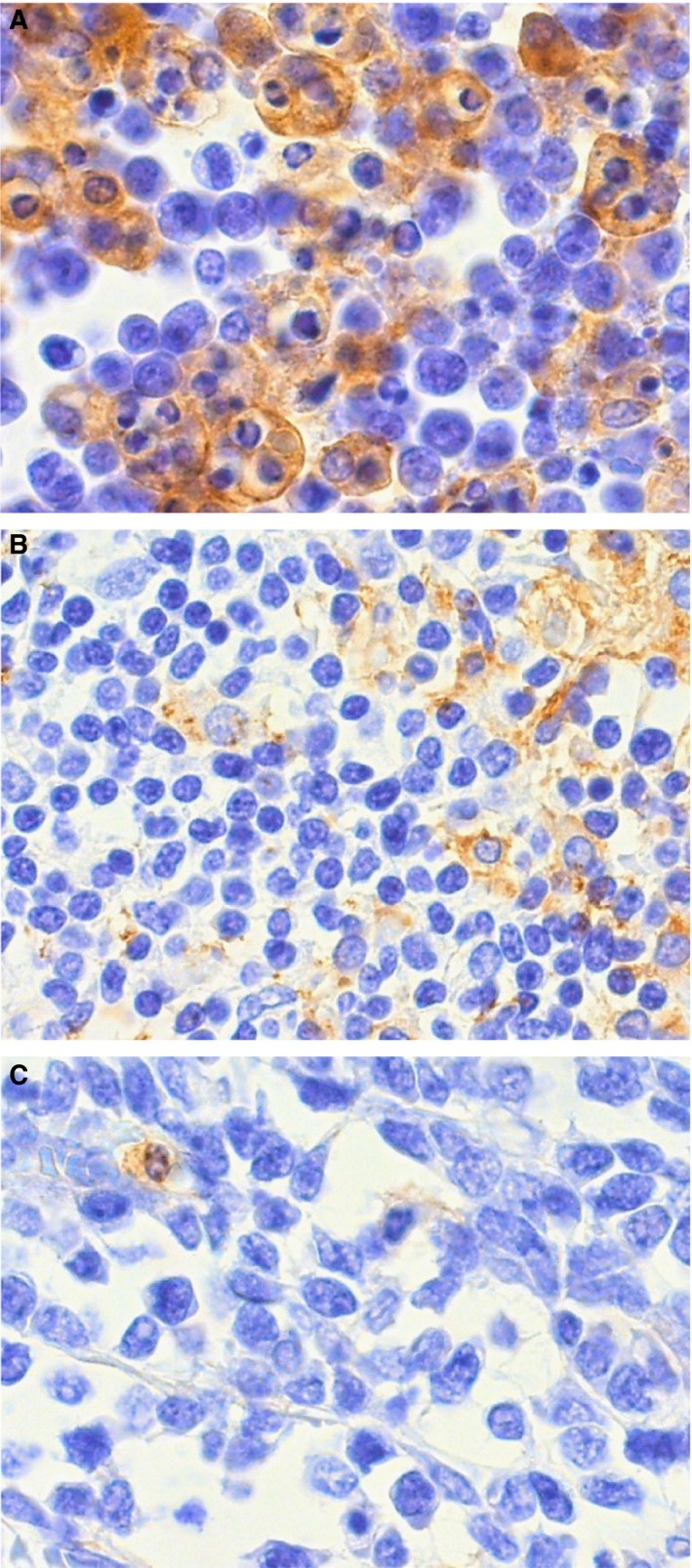
The expression pattern of PD‐L1 on microenvironment immune cells. A, High PD‐L1 expression (≥40%, miPD‐L1^high^); B, Intermediate PD‐L1 expression (5%‐40%, miPD‐L1^int^); C, Low PD‐L1 expression (<5%, miPD‐L1^low^)

**Figure 4:**
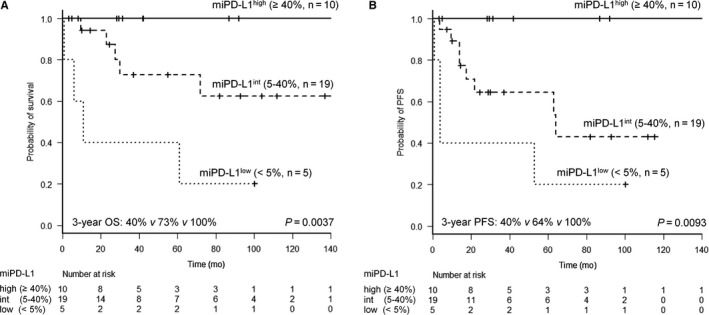
Kaplan‐Meier curves of iDLBCL patients without any EBV association, CD5 positivity, or neoplastic PD‐L1 expression. (A) Overall survival and (B) progression‐free survival based on area of PD‐L1 expression on microenvironment immune cells. Three groups with the low, intermediate, and high PD‐L1 density on microenvironment immune cells were defined by cutoff of <5% (miPD‐L1^low^), 5%‐40% (miPD‐L1^int^), and ≥40% (miPD‐L1^high^)

Thirty‐nine (70%) of the 56 nPD‐L1^−^ patients expressed PD‐L1 on ≥20% of microenvironment immune cells. Outcome according to the density of microenvironment immune cells expressing PD‐L1 was compared in iDLBCL cases treated with rituximab‐containing chemotherapy. The EBV^+^ iDLBCL, CD5‐positive iDLBCL, and/or iDLBCL with neoplastic PD‐L1 expression (n = 14) were excluded because of their more aggressive behavior than the others (*P* = 0.017, Figure 1C). Three groups with the low (miPD‐L1^low^, n = 5), intermediate (miPD‐L1^int^, n = 19), and high (miPD‐L1^high^, n = 10) PD‐L1 density on microenvironment immune cells were defined by cutoff of <5%, 5%‐40%, and ≥40%, with the favorable group having the highest PD‐L1^+^ density (Figure 3). OS was significantly different for the miPD‐L1^low^, miPD‐L1^int^, and miPD‐L1^high^ groups (*P* = 0.0037, Figure 4A) with a 3‐year OS of 40%, 73%, and 100%, respectively. PFS was also significantly different (*P* = 0.0093, Figure 4B).

## 4 DISCUSSION

The present study aimed to clarify the prognostic significance of EBV, CD5, and PD‐L1 expression associated with primary iDLBCL to facilitate patient selection for clinical trials in the era of checkpoint inhibition. Extranodal involvement has been considered a poor prognostic factor in patients with DLBCL.^35^ Multivariate analysis of nodal and extranodal DLBCL revealed that patients with small intestinal involvement had worse OS in the rituximab era.^5^ Previous studies have shown that surgery combined with chemotherapy and gene translocation involving the immunoglobulin heavy chain is associated with improved outcome in primary intestinal DLBCL,^36^, ^37^ whereas perforation and age ≥65 years are associated with worse prognosis.^37^, ^38^ However, the biological properties or clinicopathological heterogeneity of primary iDLBCL has not been addressed well in the past because of the paucity of well‐documented cases. Although the recent development of endoscopic technology and other diagnostic tools enables us to assess this rare disease, the clinical distinctiveness is unproven. Here, we shed light on the heterogeneity of primary iDLBCL including EBV^+^ and de novo CD5^+^ types and further demonstrated that miPD‐L1 expression (≥20%) is an independent prognostic factor for better survival, whereas EBER positivity and nPD‐L1 expression (≥5%) are independent prognostic factors for poor survival. Moreover, we documented the unique clinicopathological characteristics of EBV^+^ iDLBCL found, primarily among immunodeficiency‐associated patients, reflecting the vulnerability of the small and large intestines to immunologically deteriorating processes. The PD‐1/PD‐L1 immune checkpoint has been attracting attention in research on various cancers.^18^, ^19^ In addition, recent studies have suggested a link between PD‐L1 expression and EBV infection.^12^, ^17^, ^25^ Our results may be useful for selecting patients in trials in the immune‐oncology era.

In our series, EBV^+^ iDLBCL resulted in significantly worse survival than EBV^−^ iDLBCL and was identified as an independent prognostic indicator in the multivariate analysis. Most cases of EBV^+^ DLBCL exhibit activation of nuclear factor‐κB and Janus kinase‐signal transducer and activator of transcription‐related gene (JAK/STAT) pathways and usually have an activated/non‐germinal center B‐cell immunophenotype (ABC/non‐GCB).^10^, ^11^, ^12^ Most of our cases (90%) of EBV^+^ iDLBCL had non‐GCB immunophenotype, which may be related to their adverse outcome. Our patients with EBV^+^ iDLBCL were accompanied by multiple intestinal lesions (n = 5) significantly more frequently than EBV^−^ ones, which might be suggestive of hallmark of EBV^+^ cases. In addition, seven of our 10 cases with EBV^+^ iDLBCL occurred in patients with compromised immune systems treated lymphoma‐associated immunodeficiency (peripheral T‐cell lymphoma [n = 2], classic Hodgkin lymphoma [n = 2]), and iatrogenic immunodeficiency (methotrexate [n = 1], infliximab [n = 1], and tacrolimus [n = 1]). These cases indicate that EBV^+^ iDLBCL preferably arises among patients with previous history of lymphoma or immunosuppressive drug treatment and is less common in immunocompetent individuals. These findings were not replicated in our recent series of 25 EBV^+^ gastric DLBCL cases.^14^ The striking features of EBV^+^ iDLBCL might highlight the specificity of the intestine as a preferred anatomical site affected by immunodeficiency‐associated LPDs. No previous report in the English literature has yet addressed this issue. Interestingly, the high association of EBV^+^ iDLBCL with the immunological deterioration process appeared to bear similarities with EBV^+^ DLBCL of the CNS in pathogenesis, the latter of which is associated with HIV, post‐transplantation, and iatrogenic immunodeficiency.^40^, ^41^ This analogy between the pathogenesis of primary intestinal and CNS EBV^+^ DLBCL has not been elucidated and should be clarified in the future.

The EBV^+^ iDLBCL cases arising in the setting of immune suppression evoked the possibility of an EBV^+^ mucocutaneous ulcer (EBVMCU), characterized by a self‐limited, indolent course that generally responds well to conservative management.^42^ Only one (case #10) of our 10 patients with EBV^+^ iDLBCL had localized stage (Lugano II1) and was alive with no evidence of disease at 41 months. Two ulcerative intestinal lesions were found by chance in this patient during gastric resection for carcinoma and can be regarded as EBVMCU despite its deeper invasion of the serosa.

PD‐L1 expression is considered to be a hallmark of EBV‐associated LPDs, including EBV^+^ plasmablastic lymphoma and EBV^+^ post‐transplant LPDs.^17^, ^25^ In addition, PD‐L1 induction was previously reported to be dependent on constitutive signaling through the EBV‐encoded latent membrane protein (LMP)‐1 via its effects on both the PD‐L1 enhancer and promoter, augmenting PD‐L1 expression.^43^ The positive percentage (20%) of nPD‐L1 expression in our EBV^+^ iDLBCL cases was similar to the percentage (15.6%) reported in a larger series (n = 1253).^44^ However, this contrasts with the higher incidence of nPD‐L1 expression reported in young EBV^+^ DLBCL patients without immunodeficiency (76% of patients express PD‐L1 on DLBCL cells).^12^ This discrepancy was assumed to be related to the difference between those two cohorts: young vs elderly onset age, nodal vs intestinal site, and immunocompetent vs immunodeficiency‐associated patients. In addition, Chen et al reported more frequent upregulation of PD‐L1 on tumor cells in EBV^+^ DLBCL and EBV^+^ post‐transplant LPDs (100% and 60% of cases, respectively).^17^ One potential explanation for the discrepancy with our data is the use of different antibodies. A recent study showed that the SP142 clone, which we used in this study, exhibits fewer stained tumor cells than the other clones for PD‐L1 antibody (22C3, 28‐8, and SP263), but it was tested in non–small‐cell lung cancer.^45^ Therefore, we additionally immunostained EBV^+^ iDLBCL with E1J2J and 28‐8 antibodies, which resulted in no difference from the SP142 clone data. This issue should be clarified in the future.

Of three iDLBCL cases with neoplastic PD‐L1 expression, two were simultaneously EBV‐positive. The other case (case #1) was EBV‐negative and had morphology similar to that of a giant cell‐rich variant of de novo CD5^+^ DLBCL despite its CD5 negativity on the FFPE section. In this patient cohort, the expression of PD‐L1 on tumor cells was considered an independent adverse factor. Our results are similar to those reported by Kiyasu et al.^44^ The adverse outcome associated with PD‐L1 expression on iDLBCL cells was suggested to be caused by the activity of the PD‐1/PD‐L1 pathway. Some clinical studies have shown responses to PD‐1 blockade in patients with PD‐L1 expression on tumor cells or on tumor‐infiltrating immune cells.^22^, ^23^ As patients with nPD‐L1^+^ iDLBCL are characterized by an aggressive clinical course in the rituximab era, they represent good candidates for novel therapies that enhance antitumor immune responses. However, whether patients with EBV^+^ iDLBCL express PD‐L1 on microenvironment immune cells, but not on tumor cells, should be clarified in future studies.

In our patients with iDLBCL, miPD‐L1‐negativity (<20%) was a poor independent prognostic factor for survival. EBV^+^ iDLBCL and nPD‐L1^+^ cases were characterized by an aggressive clinical course in this series. In addition, patients with de novo CD5^+^ DLBCL tended to have a worse prognosis than CD5‐negative cases, which is in agreement with Yamaguchi et al^46^ In our previous report on primary gastric DLBCL (gDLBCL), miPD‐L1‐negativity (<20%) and CD5 positivity were not confirmed to have poorer outcomes in contrast to iDLBCL, whereas EBV harboring on tumor cells was an independent adverse factor in both the gDLBCL and iDLBCL series.^14^ Notably, none of the gDLBCL cases examined in our series expressed PD‐L1 on tumor cells. These findings suggest the clinical and biological distinctiveness of iDLBCL from gDLBCL, though it is too early to draw firm conclusions from the present data.

Among 34 iDLBCL cases, except for CD5^+^, EBV^+^, and/or nPD‐L1^+^ cases, patients in the miPD‐L1^high^ group had an extremely better prognosis, with a 3‐year PFS and OS of 100%. In contrast, patients in the miPD‐L1^low^ group had extremely poor outcomes. The present data indicate that PD‐L1 expression on tumor cells or microenvironment immune cells had an opposite prognostic impact among our patients. This issue was recently noted by Miyoshi et al and Asano et al among patients with ATLL.^47^, ^48^ Some clinical trials have reported that the tumor microenvironment generally correlates with higher response rates to anti‐PD‐1/PD‐L1 therapies.^22^, ^49^ However, the administration of immunotherapy against the PD‐1/PD‐L1 pathway for iDLBCL with high PD‐L1 expression in the microenvironment may be considered carefully due to their extremely favorable outcome, with the exception of CD5^+^, EBV^+^, and nPD‐L1^+^ cases.

In summary, EBV^+^ iDLBCL characterized by poor outcome mainly comprises immunodeficiency‐associated patients, which has not been documented previously. In addition, EBV^+^ cases were most likely to express PD‐L1 on tumor cells or microenvironment immune cells, and nPD‐L1^+^ iDLBCL was characterized by an aggressive clinical course. Therefore, we recommend routinely evaluating EBV and PD‐L1 in patients with iDLBCL to provide a better assessment of prognosis and select candidates for immune checkpoint inhibitors. However, among iDLBCL patients without any EBV association, CD5 positivity, or neoplastic PD‐L1 expression, the administration of immunotherapy against the PD‐1/PD‐L1 pathway for iDLBCL patients with high PD‐L1 expression on microenvironment immune cells should be considered carefully due to their extremely favorable outcome. The prognostic impact of PD‐L1 may be diverse in different diseases, which should be examined in future studies.

## CONFLICT OF INTEREST

The authors have no significant relationships with or financial interests in any commercial companies pertaining to this article.
