# Supplementary material for: Clinicopathological analysis of primary intestinal diffuse large B‐cell lymphoma: Prognostic evaluation of CD5, PD‐L1, and Epstein‐Barr virus on tumor cells
Source: Cancer Med. 2018 Nov 18;7(12):6051–63. doi: 10.1002/cam4.1875 (PMC6308116; doi:10.1002/cam4.1875)
Supplement: Supplementary file 1 [file CAM4-7-6051-s001.docx]

| **TABLE S1. Antibodies used in the immunophenotypic analysis of iDLBCL** | | | |
| --- | --- | --- | --- |
| Antigen | Clone | Dilution | Source |
| CD3 | F7.238 | 1:30 | Dako |
| CD10 | 56C6 | 1:40 | Dako |
| CD20 | L26 | 1:1 | Dako |
| CD30 | Ber-H2 | 1:20 | Dako |
| CD79a | JCB117 | 1:80 | Dako |
| Bcl-2 | l24 | 1:80 | Dako |
| Bcl-6 | LN22 | 1:20 | Novocastra |
| MUM1/IRF4 | MUM1P | 1:200 | Santa Cruz Biotechnology |
| PD-L1 | SP142 | 1:50 | Spring Bioscience |
| LMP1 | C-S.1-4 | 1:100 | Dako |
| EBNA2 | PE2 | 1:50 | Dako |
